# Long-term, open-label, phase 3 study of rasagiline in Japanese patients with early Parkinson’s disease

**DOI:** 10.1007/s00702-018-1964-3

**Published:** 2019-01-28

**Authors:** Nobutaka Hattori, Atsushi Takeda, Shinichi Takeda, Akira Nishimura, Tadayuki Kitagawa, Hideki Mochizuki, Masahiro Nagai, Ryosuke Takahashi

**Affiliations:** 1 Department of Neurology, Juntendo University Faculty of Medicine, 2-1-1, Hongo, Bunkyo-ku, Tokyo, 113-8421 Japan; 2 Department of Neurology, National Hospital Organization Sendai Nishitaga Hospital, Miyagi, Japan; 3 Japan Development Center, Takeda Pharmaceutical Company Limited, Osaka, Japan; 4 Department of Neurology, Osaka University Graduate School of Medicine, Osaka, Japan; 5 Clinical Therapeutic Trial Center, Ehime University Hospital, Ehime, Japan; 6 Department of Neurology, Kyoto University Graduate School of Medicine, Kyoto, Japan

**Keywords:** Clinical trial, Japanese, Long term, Parkinson’s disease, Rasagiline

## Abstract

Rasagiline is a monoamine oxidase B inhibitor with demonstrated efficacy and safety in patients with Parkinson’s disease (PD). We recently conducted the first randomized, double-blind, placebo-controlled trial of rasagiline in Japanese patients with early PD and now report the results of its open-label extension (clinicaltrials.gov, NCT02337751). In the double-blind trial, patients aged 30–79 years with PD diagnosis within 5 years and Movement Disorder Society-Unified Parkinson’s Disease Rating Scale (MDS-UPDRS) Part II + Part III total score ≥ 14 were randomized to placebo or rasagiline 1 mg/day for 26 weeks. Of 210 patients who completed the randomized trial, 198 (95 placebo, 103 rasagiline) entered the extension and received rasagiline 1 mg/day for 26 weeks. Analyses included patients who received rasagiline anytime during double-blind and/or extension periods; mean (standard deviation) treatment duration was 169.6 (39.57) and 316.5 (88.89) days in placebo–rasagiline (*n* = 95) and rasagiline–rasagiline (*n* = 117) groups, respectively. The incidence of treatment-emergent adverse events (TEAEs; primary outcome) was 53.7% and 77.8% in the placebo–rasagiline and rasagiline–rasagiline groups, respectively. Drug-related TEAEs occurred in 24.2% and 49.6% of patients and serious TEAEs occurred in four (two drug related) and six (one drug related) patients in the placebo–rasagiline and rasagiline–rasagiline groups, respectively. The mean change in MDS-UPDRS Part II + III total score from baseline (before rasagiline) was − 2.8 points in both the placebo–rasagiline (mean [95% confidence interval] − 2.8 [− 4.05, − 1.59]) and rasagiline–rasagiline (− 2.8 [− 4.57, − 1.01]) groups. In conclusion, up to 52 weeks, rasagiline was well tolerated with sustained motor symptom improvement, supporting its use in Japanese patients with early PD.

## Introduction

Parkinson’s disease (PD) is a neurodegenerative disorder caused by the progressive loss of dopaminergic neurons in the substantia nigra (National Collaborating Centre for Chronic Conditions (UK) 2006). Although PD is primarily a movement disorder that is characterized by tremor, rigidity, postural instability, and bradykinesia, patients with PD also exhibit non-motor symptoms, including depression, anxiety, cognitive changes, and sleeping problems (National Collaborating Centre for Chronic Conditions (UK) 2006). The incidence of PD increases with age, and its prevalence has risen in recent years (Pringsheim et al. 2014; Hirsch et al. 2016; Savica et al. 2016). A recent meta-analysis of 47 international studies reported prevalence rates ranging from 41 per 100,000 in people aged 40–49 years to 1903 per 100,000 in people aged over 80 years (Pringsheim et al. 2014). In Japan, a country with a rapidly aging population, the prevalence of PD is estimated to be between 100 and 150 per 100,000 (Japan Intractable Diseases Information Center 2015).

Current symptomatic treatments for PD target the affected dopaminergic systems by replacing dopamine with levodopa or dopamine agonists, or by inhibiting dopamine metabolism (monoamine oxidase B [MAOB] inhibitors) (Rizek et al. 2016). Levodopa is the standard treatment for PD; however, its long-term use is associated with motor complications, such as dyskinesia and motor fluctuations (Tsouli and Konitsiotis 2010; Finberg and Rabey 2016; Rizek et al. 2016). Dopamine agonists have more favorable pharmacokinetics than levodopa, including a longer half-life that provides a less pulsatile dopaminergic stimulation of the striatum, and their use can delay levodopa treatment, thereby reducing motor complications; however, side effects are common (Tsouli and Konitsiotis 2010). Although MAOB inhibitors have more modest effects on parkinsonian symptoms than levodopa, they are well tolerated and the relative lack of side effects is an advantage when treating early PD (Robakis and Fahn 2015).

The efficacy and safety of the MAOB inhibitor rasagiline have been demonstrated in several randomized controlled trials, both as monotherapy in early PD (Parkinson Study Group 2002, 2004; Olanow et al. 2009) and as adjunct to levodopa in advanced PD (Parkinson Study Group 2005; Rascol et al. 2005). In patients with early PD, the TEMPO (Parkinson Study Group 2002, 2004) and ADAGIO (Olanow et al. 2009) studies demonstrated that 1 mg/day rasagiline improved symptoms, as assessed by Unified Parkinson’s Disease Rating Scale (UPDRS) scores, compared with placebo over 26 or 36 weeks of treatment. Both studies suggested that starting rasagiline treatment early was possibly advantageous over delayed-start treatment (Parkinson Study Group 2004; Olanow et al. 2009). Open-label extensions of the TEMPO and ADAGIO studies confirmed that rasagiline is effective and well tolerated for up to 6.5 years (Hauser et al. 2009; Lew et al. 2010; Rascol et al. 2016). However, the TEMPO and ADAGIO studies primarily recruited patients from the United States and Europe, and no patients from Asia were included (Parkinson Study Group 2002, 2004; Olanow et al. 2009).

We recently conducted the first phase 3, randomized, placebo-controlled trial of rasagiline in Japanese patients with early PD (Hattori et al. 2018), which was also the first to use the more sensitive Movement Disorder Society-Unified Parkinson’s Disease Rating Scale (MDS-UPDRS) (Goetz et al. 2008). Rasagiline (1 mg/day) for 26 weeks was associated with a significantly greater improvement in combined MDS-UPDRS Part II and Part III (Part II + III) total score, compared with placebo, with no safety concerns (Hattori et al. 2018). We now report the results of an open-label extension study of this randomized trial. The aim of the extension study was to assess the long-term (up to 52 weeks) safety and efficacy of rasagiline (1 mg/day) in Japanese patients with early PD. In addition, documenting data obtained over 12 months of rasagiline treatment is required to fulfill regulatory requirements for marketing authorization in Japan.

## Methods

### Ethics

This study was conducted at 68 medical institutions throughout Japan between July 2015 and March 2017. The study was approved by the institutional review board of each study site and conducted in compliance with the International Conference on Harmonisation, Good Clinical Practice guidelines, and the Declaration of Helsinki. All patients provided written informed consent. The study was sponsored by Takeda Pharmaceutical Co. Ltd., and is registered at clinicaltrials.gov (NCT02337751) and the Japan Pharmaceutical Information Center (JapicCTI-152761).

### Study design and treatment protocol

**Fig. 1:**
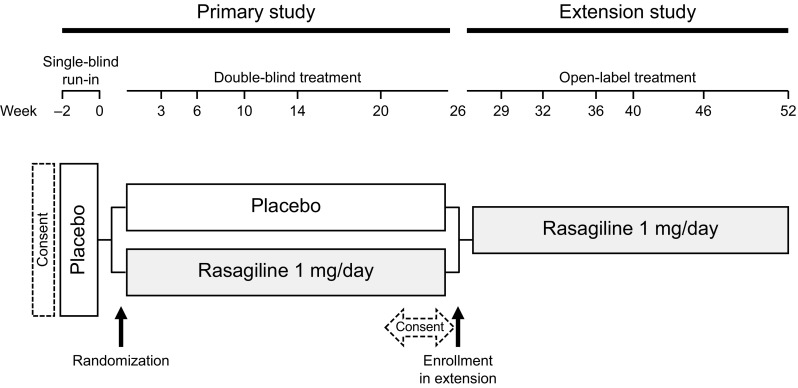
Study design

This study was a multicenter, open-label, extension study to evaluate the safety and efficacy of long-term administration of rasagiline at 1 mg/day for an additional 26 weeks in Japanese patients with early PD who completed the preceding, 26-week, randomized, double-blind, placebo-controlled, phase 3 trial (NCT02337725) (Hattori et al. 2018). In the double-blind trial, after a 2-week run-in period with placebo, patients were randomized to placebo or oral rasagiline (1 mg/day; Teva Pharmaceutical Industries Ltd.) once daily (Fig. 1). After completion of the 26-week double-blind trial, patients were invited to participate in this extension study (Fig. 1). There was no washout period before entering the extension. During the extension, all patients received rasagiline 1 mg/day for 26 weeks.

### Study population

Inclusion and exclusion criteria for the double-blind study have been described previously (Hattori et al. 2018). In brief, the study included male and female outpatients aged 30–79 years with PD diagnosed within the past 5 years, an MDS-UPDRS Part II + III total score of ≥ 14, and at modified Hoehn and Yahr stages 1–3. Patients were excluded if they had a Mini-Mental State Examination score of ≤ 24; had received selegiline within 90 days, or levodopa, dopamine agonist, amantadine, or anticholinergic medication within 30 days; or had been treated with selegiline, levodopa, or dopamine agonist for ≥ 90 days, or amantadine or anticholinergic medication for ≥ 180 days.

Patients who completed the double-blind trial without safety issues (investigators’ judgement) were eligible for inclusion in this extension study.

### Outcome measures

The primary outcome of the extension study was safety, assessed by the incidence of treatment-emergent adverse events (TEAEs) occurring after receiving rasagiline. TEAEs are reported for 26 weeks in patients who received placebo during the double-blind study and switched to rasagiline for the extension (placebo–rasagiline [PR] group) and for 52 weeks in patients who received rasagiline during both the double-blind study and the extension study (rasagiline–rasagiline [RR] group). Other safety outcomes included standard laboratory measures, vital signs, body weight, and electrocardiogram. The Medical Dictionary for Regulatory Activities (MedDRA, version 19.0) was used to code and summarize all TEAEs.

Secondary outcome measures for efficacy evaluation included the MDS-UPDRS Part II + III total score, which used a Japanese version of the MDS-UPDRS (Kashihara et al. 2014). Part II of the MDS-UPDRS assesses motor experiences of daily living (13 items) and was evaluated by the patients. Part III assesses motor examination (18 items) and was evaluated by certified investigators/subinvestigators. Each subscale is rated from 0 (normal) to 4 (severe) (Goetz et al. 2008). Other efficacy outcomes included MDS-UPDRS Part I total score (non-motor experiences of daily living; 13 items rated 0–4), Part II total score, Part III total score, and tremor, bradykinesia, and rigidity scores. We also assessed disease-specific quality of life using the patient-reported Parkinson’s Disease Questionnaire-39 (PDQ-39) summary index (Jenkinson et al. 1997).

### Statistical analysis

We aimed to enroll 182 patients into this extension study to obtain 155 patients treated with rasagiline for 26 weeks and 59 patients treated with rasagiline for 52 weeks. These sample sizes would allow the collection of sufficient safety data to meet Japanese regulatory requirements for the number of patients treated for 6 months and 12 months, respectively. The full analysis set (FAS) and the safety analysis set were identical and included randomized patients who received at least one dose of rasagiline at any time during the double-blind and/or extension studies. A last observation carried forward approach was used for end point values. Missing data were not otherwise imputed. Baseline was defined as the start of the double-blind study, except for MDS-UPDRS and PDQ-39 scores, where baseline was defined as before the first administration of rasagiline. All safety and efficacy measurements are summarized descriptively. Baseline characteristics are presented as number (%) and/or mean (standard deviation, SD). Safety data are presented as number (%). Efficacy data are presented as mean change from baseline, with SD and/or 95% confidence interval (CI). All statistical analyses were performed using SAS® version 9.2 (SAS Institute, Cary, NC, USA).

## Results

### Patient disposition

**Fig. 2:**
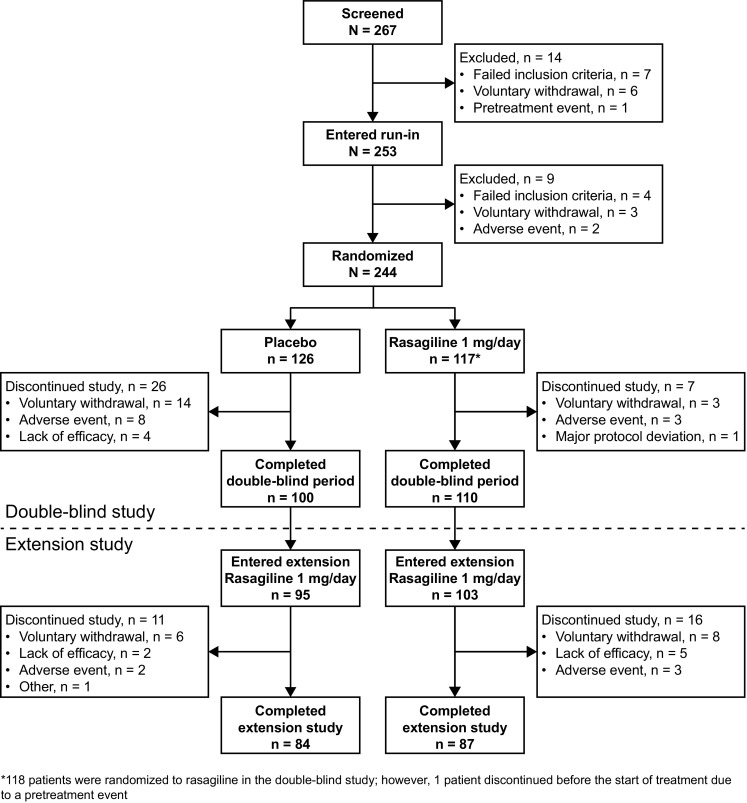
Patient disposition during the double-blind and extension studies

Of 210 patients who completed the double-blind study, 198 patients (95 placebo and 103 rasagiline) entered the extension study (Fig. 2). Of these, 171 patients completed the extension. The rate of discontinuation was similar in the PR (11 of 95; 11.6%) and RR (16 of 103; 15.5%) groups. The main reason for discontinuation from the extension study was voluntary withdrawal. The rate of discontinuation due to an adverse event was similar in each 26-week period of rasagiline treatment (2/95 [2.1%] in the PR group and 3/117 [2.6%] in the RR group during the first 26-week period; 3/103 [3.0%] in the RR group during the second 26-week period). The FAS and the safety analysis set included 212 patients (95 in the PR group, 117 in the RR group) who received rasagiline at any time during the double-blind and/or extension studies. Three patients (one in the PR group, two in the RR group) did not have post-baseline data for some efficacy end points and were not included in those efficacy analyses.

### Baseline characteristics

Patient characteristics at baseline are shown in Table 1. Slightly more than half (approximately 55%) of patients were female, the mean age was 65.4 and 67.4 years, the mean duration of PD was 1.52 and 1.97 years, and the mean MDS-UPDRS Part II + III total score, measured before the start of rasagiline treatment, was 33.0 and 34.4 in the PR and RR groups, respectively.

**Table 1:** Patient characteristics at baseline

Characteristic	Placebo–rasagiline (*n* = 95)	Rasagiline–rasagiline (*n* = 117)
Age		
Mean (SD), years	65.4 (9.13)	67.4 (8.99)
< 65 years, *n* (%)	35 (36.8)	39 (33.3)
≥ 65 years, *n* (%)	60 (63.2)	78 (66.7)
Sex, *n* (%)		
Male	42 (44.2)	53 (45.3)
Female	53 (55.8)	64 (54.7)
Duration of Parkinson’s disease		
Mean (SD), years	1.52 (1.223)	1.97 (1.981)
< 1.5 years, *n* (%)	55 (57.9)	57 (48.7)
≥ 1.5 years, *n* (%)	40 (42.1)	60 (51.3)
Modified Hoehn and Yahr stage, *n* (%)		
Mean (SD)	2.13 (0.627)	2.18 (0.628)
< 2.0	15 (15.8)	18 (15.4)
2.0 to < 3.0	58 (61.1)	65 (55.6)
≥ 3.0	22 (23.2)	34 (29.1)
MDS-UPDRS score, mean (SD)		
Part II + III total score	33.0 (15.86)	34.4 (16.95)
Part I total score	6.0 (3.66)	5.5 (3.83)
Part II total score	8.1 (5.20)	7.2 (5.47)
Part III total score	24.9 (12.94)	27.2 (13.80)
Tremor	5.4 (4.45)	5.7 (4.52)
Bradykinesia	10.8 (6.50)	12.3 (7.06)
Rigidity	5.9 (3.69)	5.9 (3.34)
PDQ-39 summary index score, mean (SD)	13.24 (10.864)	10.50 (10.042)

For age, sex, duration of Parkinson’s disease, and modified Hoehn and Yahr stage, baseline was defined as the start of the double-blind study; for MDS-UPDRS and PDQ-39 scores, baseline was defined as the start of rasagiline treatment

*MDS-UPDRS* Movement Disorder Society-Unified Parkinson’s Disease Rating Scale, *PDQ*-*39* Parkinson’s Disease Questionnaire-39, *SD* standard deviation

### Safety

The mean (SD) duration of rasagiline treatment was 169.6 (39.57) days in the PR group and 316.5 (88.89) days in the RR group. The incidences of TEAEs and drug-related TEAEs were 53.7% and 24.2%, respectively, over 26 weeks in the PR group and 77.8% and 49.6%, respectively, over 52 weeks in the RR group (Table 2). The most common TEAEs (reported by ≥ 5% of patients in either group) were nasopharyngitis, fall, eczema, headache, and contusion. Most TEAEs were mild or moderate, although three patients (one in the PR group; two in the RR group) had a severe TEAE (all were serious TEAEs).

**Table 2:** Treatment-emergent adverse events

Treatment-emergent adverse event	Placebo–rasagiline (*n* = 95) 26 weeks	Rasagiline–rasagiline (*n* = 117) 52 weeks
Any TEAE, *n* events/*n* (%) patients		
Any	94/51 (53.7)	278/91 (77.8)
Related to study drug	31/23 (24.2)	118/58 (49.6)
Severe	1/1 (1.1)	2/2 (1.7)
Leading to study drug discontinuation	1/1 (1.1)	6/6 (5.1)
Serious TEAEs, *n* events/*n* (%) patients		
Any	4/4 (4.2)	6/6 (5.1)
Related to study drug	2/2 (2.1)	1/1 (0.9)
Leading to study drug discontinuation	0	2/2 (1.7)
TEAEs by MedDRA Preferred Term, *n* patients (%)^a^		
Nasopharyngitis	17 (17.9)	31 (26.5)
Fall	6 (6.3)	11 (9.4)
Eczema	2 (2.1)	9 (7.7)
Contusion	5 (5.3)	5 (4.3)
Back pain	4 (4.2)	5 (4.3)
Headache	1 (1.1)	7 (6.0)
Dizziness	3 (3.2)	2 (1.7)
Pruritis	1 (1.1)	4 (3.4)
Arthralgia	0	4 (3.4)
Gastroenteritis	3 (3.2)	1 (0.9)
Oropharyngeal pain	0	4 (3.4)
Intervertebral disc protrusion	3 (3.2)	0

*MedDRA* Medical Dictionary for Regulatory Activities, *TEAE* treatment-emergent adverse event

^a^TEAEs occurring in ≥ 3% of patients in either group

Serious TEAEs occurred in four patients (4.2%) in the PR group and six patients (5.1%) in the RR group. In the PR group, there was one case each of mechanical ileus, osteoarthritis, prostate cancer (considered drug related), and microscopic polyangiitis (considered drug related). In the RR group, there was one case each of inguinal hernia, leukoplakia oral, abnormal hepatic function (considered drug related), femoral neck fracture, spinal compression fracture, and intracranial aneurysm. There were no deaths during the study.

Changes in laboratory parameters, vital signs, electrocardiogram, and weight were small in both study groups, and the rate of abnormal values reported as TEAEs was low (5.3% in the PR group; 6.0% in the RR group).

### Efficacy

**Fig. 3:**
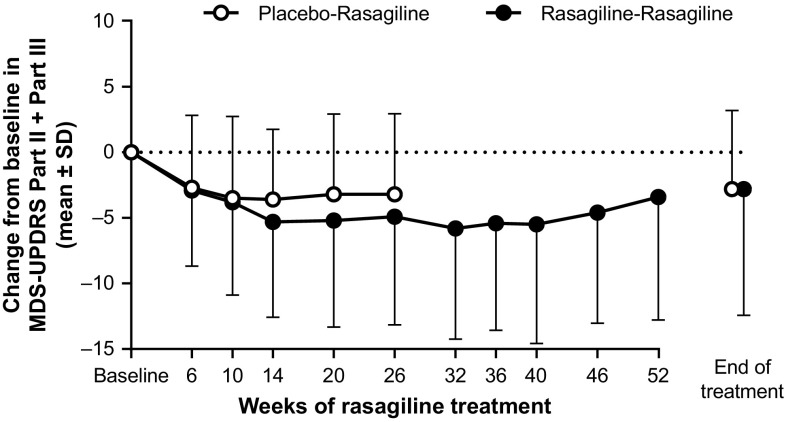
Change from baseline (before rasagiline) in combined MDS-UPDRS Part II + Part III total score for the rasagiline–rasagiline (*n* = 115) and placebo–rasagiline (*n* = 94) groups. A last observation carried forward approach was used for the end of treatment data points. *MDS-UPDRS* Movement Disorder Society-Unified Parkinson’s Disease Rating Scale, *SD* standard deviation

The MDS-UPDRS Part II + III total score decreased from baseline (before rasagiline treatment) in both PR and RR groups (Fig. 3). This decrease was evident at the first time point (6 weeks) after starting rasagiline treatment and was sustained for up to 52 weeks in the RR group (Fig. 3). The mean change from baseline at the end of rasagiline treatment was − 2.8 points in both the PR (mean [95% CI]: −2.8 [− 4.05, − 1.59]) and RR (mean [95% CI]: − 2.8 [− 4.57, − 1.01]) groups. The decrease was primarily driven by a decrease in MDS-UPDRS Part III total score in both study groups (Table 3).

**Table 3:** Change from baseline (start of rasagiline treatment) in MDS-UPDRS and PDQ-39 summary index scores at end of rasagiline treatment (last observation carried forward)

Score, change from baseline Mean (SD), [95% CI]	Placebo–rasagiline (*n* = 94) 26 weeks	Rasagiline–rasagiline (*n* = 116) 52 weeks
MDS-UPDRS		
Part I total score	− 0.2 (2.65) [− 0.74, 0.34]	0.3 (3.27) [− 0.33, 0.87]
Part II total score	0.1 (2.83) [− 0.43, 0.73]	1.5 (4.04) [0.76, 2.24]
Part III total score	−2.9 (5.08) [− 3.99, − 1.91]	− 4.0 (7.64)^a^ [− 5.44, − 2.62]
Tremor	−0.8 (2.18) [− 1.22, − 0.33]	− 1.0 (2.99) [− 1.56, − 0.46]
Bradykinesia	−1.2 (3.07) [− 1.83, − 0.57]	− 1.8 (4.16)^a^ [− 2.53, − 1.00]
Rigidity	− 0.7 (1.65) [− 1.02, − 0.34]	− 0.8 (2.22)^a^ [− 1.22, − 0.40]
PDQ-39 summary index	−1.50 (6.599) [− 2.849, − 0.146]	2.86 (8.509)^a^ [1.286, 4.430]

Data are shown for patients in the FAS with no missing values

*CI* Confidence interval, *FAS* full analysis set, *MDS-UPDRS* Movement Disorder Society-Unified Parkinson’s Disease Rating Scale, *PDQ-39* Parkinson’s Disease Questionnaire-39, *SD* standard deviation

^a^*n* = 115

The MDS-UPDRS Part I score did not change substantially in either study group (Table 3). The Part II score did not change substantially in the PR group, but increased in the RR group, most likely because of natural disease progression during the longer observation period for this group (Table 3). The Part III score, as well as the tremor, bradykinesia, and rigidity scores, decreased in both study groups (Table 3).

The PDQ-39 summary index score decreased slightly in the PR group, but increased slightly in the RR group (Table 3), again, most likely because of natural disease progression (Grosset et al. 2007).

## Discussion

This is the first phase 3 study to demonstrate the safety and efficacy of long-term rasagiline monotherapy in Japanese patients with early PD. Treatment with rasagiline (1 mg/day) was well tolerated for up to 52 weeks, with no safety concerns. Sustained improvements in MDS-UPDRS Part II + III total scores were seen in patients who received rasagiline for 52 weeks, as well as in patients who received rasagiline for 26 weeks. These results support the safety and efficacy of rasagiline monotherapy as a long-term treatment option in Japanese patients with early PD.

Rasagiline monotherapy was well tolerated for up to 52 weeks in Japanese patients with early PD. In the preceding randomized trial, the incidence of TEAEs, drug-related TEAEs, serious TEAEs, and TEAEs leading to discontinuation was 62.4%, 39.3%, 3.4%, and 2.6%, respectively, in patients treated with rasagiline for 26 weeks (Hattori et al. 2018). In the current 52-week extension study, the incidence of these TEAEs increased in proportion to the duration of treatment. In addition, the rate of discontinuation due to an adverse event was low and did not increase with prolonged treatment, indicating that rasagiline was well tolerated. Other than nasopharyngitis, all individual TEAEs occurred at an incidence < 10%, even over 52 weeks of rasagiline treatment. The type and frequency of TEAEs were similar to those reported in the TEMPO and ADAGIO studies (Parkinson Study Group 2002, 2004; Olanow et al. 2009; Lew et al. 2010; Rascol et al. 2016), indicating that there were no new safety issues or issues specific to Japanese patients. In the rasagiline 1 mg/day group of the TEMPO study, the incidence of adverse events was 79.5% during the first 26 weeks of treatment, and 66.4% during the subsequent 26 weeks of treatment (Parkinson Study Group 2004). The incidence of adverse events during the total 52-week rasagiline treatment period was not reported for the TEMPO study; however, the incidence of TEAEs in the RR group at 26 weeks in the randomized trial (62.4%) and at 52 weeks in this extension study (77.8%) is similar to the incidences reported in the rasagiline 1 mg/day group of the TEMPO study. These results confirm the favorable safety profile of long-term rasagiline use in Japanese patients with early PD.

Rasagiline is mainly metabolized by CYP1A2, which is known to exhibit no major ethnic differences. In addition, similar pharmacokinetic profiles have been reported between Japanese and Caucasian populations for rasagiline and its main metabolite (Elgardt et al. 2018).

Consistent with previous studies (Parkinson Study Group 2002; Hauser et al. 2009; Olanow et al. 2009), monotherapy with 1 mg/day rasagiline improved the signs and symptoms of PD in Japanese patients, as assessed by MDS-UPDRS scores. At the end of rasagiline treatment, the MDS-UPDRS Part II + III total score improved from baseline (before rasagiline) by a mean of − 2.8 points in both groups. This change from baseline was less than that seen with rasagiline at the end of the preceding 26-week double-blind trial (mean change from baseline = − 4.52 points) (Hattori et al. 2018), most likely due to the continued progression of PD in these patients during the long-term extension study. As in the preceding double-blind trial (Hattori et al. 2018), the largest improvement in individual MDS-UPDRS subscores was seen in the Part III motor examination score. A change in MDS-UPDRS Part III of − 3.25 points has been suggested as the minimal clinically important difference for improvement (Horváth et al. 2015); in this study, patients treated for 52 weeks achieved this level (− 4.0 points), whereas patients treated for 26 weeks approached this level (− 2.9 points). Improvements in the MDS-UPDRS tremor, bradykinesia, and rigidity scores were also seen in both groups, further supporting the long-term efficacy of rasagiline for the improvement of motor symptoms in patients with early PD.

This is the first long-term study of an MAOB inhibitor using the newer MDS-UPDRS. Refinements in the conventional UPDRS by the Movement Disorder Society led to a more comprehensive, more sensitive, and more consistent system for scoring motor and non-motor symptoms of PD (Goetz et al. 2008). The MDS-UPDRS includes items that are not covered by the original UPDRS, including items in Part III such as freezing of gait (Goetz et al. 2008). In addition, the MDS-UPDRS is more sensitive than the UPDRS for detecting changes at the milder end of the spectrum (Goetz et al. 2008), which is particularly useful when assessing treatment efficacy in patients with early PD, as in the current study. The instructions that accompany the MDS-UPDRS, including the Japanese version (Kashihara et al. 2014), are clearer and more detailed than in the original UPDRS, which results in greater consistency across multiple study sites (Goetz et al. 2008).

As mentioned, this extension study is the first large, long-term study of an MAOB inhibitor in Japanese patients to be reported and the first to use MDS-UPDRS instead of UPDRS scores. However, interpretation of the results is limited by its open-label design, the lack of a placebo or active comparator group, and the treatment period of up to 52 weeks.

In conclusion, rasagiline monotherapy (1 mg/day) for up to 1 year was well tolerated in Japanese patients with early PD and led to sustained improvements in symptoms, as assessed by MDS-UPDRS scores. These results support the use of rasagiline as a long-term early treatment option for patients with PD in Japan.
